# Clinical use of pharmacogenomics

**DOI:** 10.1186/1742-4690-9-S1-I2

**Published:** 2012-05-25

**Authors:** Amalio Telenti

**Affiliations:** 1 University of Lausanne, Switzerland

## 

The expanding list of antiretroviral drugs, the increasing possibilities in therapy of HCV and other co-infections represent an attractive setting for individualized prescription. However, there has been considerable hype, and limited impact of pharmacogenetics in care.

The development of pharmacogenetic tools would be of interest considering the complexity of treatment, the cost, and the expectation of long-term exposure. It would also be desirable given the possibility of serious toxicity, but also treatment intolerance, cumulative toxicity or the unfavorable interaction of drugs and aging, metabolic, cardiovascular and bone disease processes.

This presentation will review available tests, the position of agencies on the inclusion of pharmacogenetic information in labels, and a perspective on developments in genomic medicine.

